# Diffusion and dissemination of evidence-based dietary srategies for the prevention of cancer

**DOI:** 10.1186/1475-2891-4-13

**Published:** 2005-04-08

**Authors:** Donna Ciliska, Paula Robinson, Tanya Armour, Peter Ellis, Melissa Brouwers, Mary Gauld, Fulvia Baldassarre, Parminder Raina

**Affiliations:** 1 School of Nursing, McMaster University, 1200 Main St. W. Hamilton, Ontario, L8N 3Z5, Canada; 2 Department of Clinical Epidemiology & Biostatistics (CEB), McMaster University, 1200 Main St. W. Hamilton, Ontario, L8N 3Z5, Canada; 3 Cancer Care Ontario Program in Evidence Based Care (CCO PEBC) McMaster University, 50 Main St. E. Hamilton, Ontario, L8N 1E9, Canada; 4 Hamilton Regional Cancer Centre, 699 Concession Street, Hamilton, Ontario, L8V 5C2, Canada; 5 McMaster University Evidence-based Practice Center, 50 Main St. E. Hamilton, Ontario, L8N 1E9, Canada

## Abstract

**Objective:**

The purpose was to determine what strategies have been evaluated to disseminate cancer control interventions that promote the uptake of adult healthy diet?

**Methods:**

A systematic review was conducted. Studies were identified by searching MEDLINE, PREMEDLINE, Cancer LIT, EMBASE/Excerpta Medica, PsycINFO, CINAHL, the Cochrane Database of Systematic Reviews, and reference lists and by contacting technical experts. English-language primary studies were selected if they evaluated the dissemination of healthy diet interventions in individuals, healthcare providers, or institutions. Studies of children or adolescents only were excluded.

**Results:**

One hundred one articles were retrieved for full text screening. Nine reports of seven distinct studies were included; four were randomized trials, one was a cohort design and three were descriptive studies. Six studies were rated as methodologically weak, and one was rated as moderate. Studies were not meta-analyzed because of heterogeneity, low methodological quality, and incomplete data reporting. No beneficial dissemination strategies were found except one that looks promising, the use of peer educators in the worksite, which led to a short-term increase in fruit and vegetable intake.

**Conclusions and Implications:**

Overall, the quality of the evidence is not strong and is primarily descriptive rather than evaluative. No clear conclusions can be drawn from these data. Controlled studies are needed to evaluate dissemination strategies, and to compare dissemination and diffusion strategies with different messages and different target audiences.

## Background

It has been estimated that one-third of all cancer mortality in the United States (US) is related to diet[1]. Reviews of dietary studies have led groups, such as the American Institute for Cancer Research, to recommend that diet should largely be based on plant products with 400 grams of vegetables and fruits to provide more than 10 percent of energy consumed daily[2,3]. The American Cancer Society added that intake of high-fat foods and alcohol should be limited[4]. The national objectives in both the US and Canada have been set at five or more servings per day of fruits and vegetables[5]. Average intake falls considerably short of this. In the US, intake is estimated to be 3.4 total servings of fruits and vegetables per day on average, but differs by age, ethnicity, and socioeconomic status[6].

Considerable recent research has focused on dietary change to increase fruit and vegetable consumption and to reduce fat consumption. The effectiveness of these interventions has been the subject of several systematic reviews[7].

There is some evidence that physician education in dietary counseling is an effective dietary intervention. However, there is no consistent evidence of effectiveness of other healthcare provider directed interventions. Interventions directed at individuals that were shown to have some effect in producing dietary change include: tailored interventions; multiple interventions; and provision of multiple contacts and environmental interventions. Media campaigns may result in increased knowledge and awareness of behaviors to reduce risks[7].

As the evidence grows for the effectiveness of dietary interventions, it is expected that more attention will be given to the dissemination and diffusion of these interventions to promote dietary change. The theoretical background for research dissemination and diffusion is complex and often contradictory. There are theoretical bases and models for dissemination and diffusion of research generally, and for behavior change of healthcare practitioners and the general public. These major fields of dissemination/diffusion and practitioner/client behavior change are inconsistently integrated into the development of interventions, and the field of cancer control is no exception. Closing the gap from knowledge generation to use in decision-making for practice or policy is conceptually and theoretically hampered by diverse terms and inconsistent definitions of terms, including diffusion, dissemination, knowledge transfer or translation or uptake or utilization, adoption, and implementation. There is a lack of distinction in the research between interventions to change behavior and strategies to disseminate that information. Furthermore, many studies have combined evaluation of both interventions and strategies within one study. Some activities (e.g., media campaigns, opinion leaders, and peer educators) can be characterized as both cancer control interventions and strategies to disseminate cancer control interventions to target audiences. This can lead to confusion about what is considered a cancer control intervention and what is considered dissemination of cancer control interventions. For the purpose of this evidence report, if an activity was used to provide educational information about the benefits of a desired cancer control behavior, it was classified as a cancer control intervention. If the activity was used to provide information about the availability or benefits of a cancer control intervention, it was classified as a strategy to disseminate a cancer control intervention.

In keeping with Lomas' views, this evidence report uses the term "dissemination " to refer to the active process of transferring cancer control interventions to target audiences and "diffusion" is used to refer to the passive spread of cancer control interventions[8].

## Methods

The following question was addressed by this review: *What strategies have been evaluated to disseminate cancer control interventions that promote the uptake of adult healthy diet? *Primary studies of dissemination and diffusion strategies of dietary interventions were systematically reviewed. This review does not include studies of effectiveness of direct interventions to change dietary intake; rather, it includes those studies focused on dissemination of interventions, to adults and healthcare professionals.

Primary studies were considered for inclusion if they were English language, published ≥ 1980 and evaluated dissemination of a cancer control intervention in one of the five topic areas. All primary studies regardless of study designs were eligible for inclusion. Reports exclusively focused on children or adolescents were excluded.

Search strategies were developed as an iterative process in consultation with the McMaster Evidence based Practice Centre (EPC) librarian. The search strategy can be located at , report name Cancer Control Interventions, Diffusion and Dissemination, file name 27appc.doc. Similar databases were searched for both objectives:

- MEDLINE, the U.S. National Library of Medicine (NLM) database

- PreMedline

- CancerLIT

- EMBASE the Excerpta Medica Database

- PsychINFO

- The Cumulative Index to Nursing and Allied Health Literature (CINAHL)

- Sociological Abstracts

- HealthSTAR

- Cochrane Database of Systematic Reviews (CDSR)

- Reference lists of pertinent articles and reviews; and

- The use of technical experts

All data extraction forms were developed, pilot-tested, and revised by members of the local research team. Two reviewers completed data extraction independently for all reports. Any disagreements were resolved by consensus. The research team discussed differences that could not be resolved by these reviewers. Quality assessment was undertaken using standardized quality assessment tools developed by the Effective Public Health Practice Project.

Tables were constructed to describe the most salient characteristics of the eligible studies. Meta-analysis was not undertaken because there were substantial differences across the studies, in terms of study design, intervention assessed, outcome measurements, methodological quality, and completeness of data reporting. Therefore, the report represents a systematic narrative review of the existing evidence.

## Results

### Included Studies

**Figure 1:**
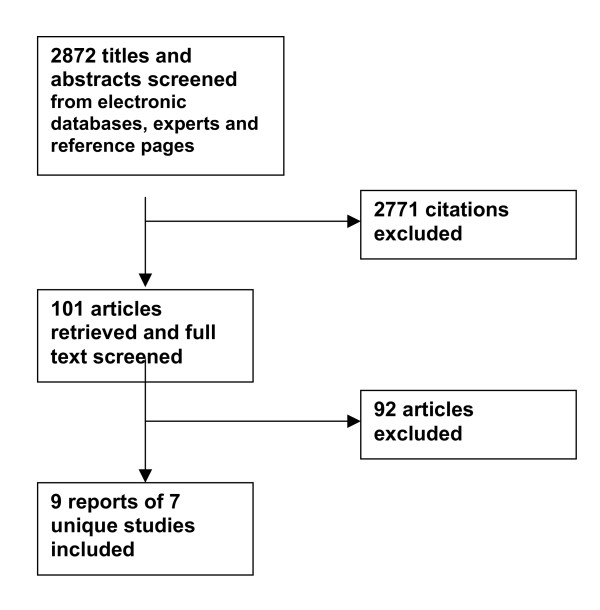
Adult Healthy Diet: Search yield for studies evaluating dissemination strategies

The electronic database search identified 2,872 articles; 101 were retrieved for full text screening (Figure 1). Of these, nine reports of seven distinct studies are included: three reports about one study [9-11] and six other studies [12-17] are presented in Evidence Table 1(see additional file 1). Ninety-two papers were excluded for lack of relevance; they did not address dissemination and diffusion strategies for dietary interventions.

Although the search inclusion criteria were broad, all of the eligible studies were conducted in the US. Six reports were published since 1998; the other four were published between 1989 and 1993[12,13,15,16]. All seven projects were funded: five by the National Cancer Institute (NCI),[9,14-17] one by the National Institute of Health (NIH),[12] and one by a private foundation[13].

One study achieved a rating of "moderate",[14] and all others were "weak" as defined by the standardized assessment tool[18]. The tool was adapted from those developed by Clarke et al.,[19]and Jadad et al[20]. As community interventions are often not evaluated by randomized trials, the tool reflects other possible study designs, and rates the following criteria: selection bias, study design, confounders, blinding, data collection methods (reliability and validity), withdrawals and dropouts, intervention integrity, and analyses. Based on a dictionary and standardized guide to assessing component ratings, each component was rated "strong," "moderate," or "weak." Content and construct validity have been established[21]. A comparison of the tool used in this review was made with the tool used in the Guide to Community Preventive Health Services[22].

Four of the studies were randomized trials[9,14,16,17,23]. None of the other studies included a comparison group; three articles were descriptive,[11,13,15] one article was a cohort study[12] (Table 1)(see additional file 1). Included studies were very diverse in the intervention that was disseminated and in strategies used for dissemination and diffusion. Only two studies compared two strategies[16,17]. Of these, one study compared the effectiveness of a training workshop to postal delivery[17]. The second study evaluated whether the use of educational facilitators (academic detailing) plus a workshop was more effective than educational facilitators (academic detailing) only[16]. Each of the other studies evaluated the effectiveness of a single dissemination strategy. One strategy assessed was "train-the-trainer" to disseminate preventive medicine education to physicians;[12] two studies evaluated media campaigns for promoting access to a phone information services;[13,15] one study assessed the effect of peer educators for improving fruit and vegetable consumption; [9-11] and one looked at the dissemination of intervention materials to control sites following the completion of a worksite nutrition intervention[14].

Outcomes were very diverse across studies and were not usually behavioral outcomes but rather process indicators, such as numbers of training sessions conducted,[12] numbers of physicians trained,[12] numbers of consumer telephone calls[13,15], counts of peer-education strategies according to gender and ethnicity,[11] and uptake of materials by control sites following an intervention[14]. Client-based outcomes included knowledge[12] and intake of fruits and vegetables[9,10].

### Dissemination Studies That Targeted Healthcare Providers

#### Train-the-trainer

One "train-the-trainer" study aimed at disseminating preventive medicine education to physicians[12]. Faculty from general internal divisions across the US were invited to apply for a month-long Stanford Faculty Development Program; 10 were chosen and trained to be Clinical Preventive Medicine facilitators. They then went to their home institutions and trained other faculty at their home site. Fidelity checks concluded that facilitators adhered closely to the curriculum they had been taught. Those medical faculty educated by the facilitators had an increase in knowledge and self-efficacy to use behavior changes to promote healthy diets. Subsequently, house staff physicians interacting with faculty who had attended the facilitator-run sessions reported an increase in the degree of preventive medicine content in teaching interactions and an increase in their ratings of self-efficacy to implement preventive medicine strategies[12]. While the train-the-trainer model shows some promise, it needs to be evaluated with a more rigorous design; furthermore, many biases are likely to be inherent in the selection of internists who were able to leave their work situation for a month of training.

#### Academic detailing (educational facilitators)

One Randomized Control Trial (RCT)[16] targeted dissemination to healthcare providers using academic detailing. In this trial by Dietrich et al., primary care medical practices were randomized to one of four groups: facilitator only, facilitator-plus-workshop, workshop only, or a control group. Practices in the facilitator-only group (n = 24) received three to four visits from a facilitator who provided detailed instruction and assistance in selecting and implementing non-computer-based office-system interventions. Practices in the facilitator-plus-workshop group (n = 26), in addition to receiving visits from an educational facilitator, had a physician from the practice attend a one-day workshop. The workshop session reviewed NCI's prevention and screening recommendations, but did not provide information on the use of office-system interventions. Practices in the workshop-only group (n = 24) attended the workshop. Practices in the control group (n = 24) received no information.

Cross-sectional patient surveys were conducted before randomization and again at 12-month follow-up. The study reported on two diet-related outcomes: (1) the number of patients reporting that their physician had advised them to reduce their fat intake and (2) the number of patients reporting their physician had advised them to increase their fiber consumption. At 12-month follow-up, significantly more eligible patients in the facilitator-only group reported their physician had advised them to reduce their fat intake compared with patients in the control group (0.56 vs. 0.47, p < 0.05). There was no significant difference in the number of patients reporting advice to decrease fat intake between the facilitator-plus-workshop group and the control group at 12-month follow-up (0.51 vs. 0.47). There was no significant increase in the number of eligible patients in the facilitator-only or facilitator-plus-workshop groups reporting advice to increase fiber consumption compared with patients in the control group at 12-month follow-up (facilitator vs. control 0.48 vs. 0.38; facilitator-plus-workshop vs. control 0.41 vs. 0.38). The overall conclusion from this RCT was that the use of educational facilitators to disseminate and implement office-system interventions could improve the provision of prevention and early detection services in community practices.

The use of educational facilitators (academic detailers) to disseminate office-system interventions appears to be a promising strategy. Further research in this area is needed.

#### Workshops

The RCT Tziraki et al.[17] assessed the effectiveness of two strategies for promoting the use of an NCI nutrition manual by primary care physicians and their office staff. The nutrition manual was modeled after the NCI publication "*How to help your patients stop smoking*". Medical practices randomized to the workshop group (n = 244) were invited to send one staff member to a three-hour training workshop on how to use the nutrition manual. Training was provided in four major components of the manual: (1) how to organize the office environment, (2) how to screen for patient adherence, (3) how to provide dietary advice, and (4) how to implement a patient follow-up system. Medical practices assigned to the postal-delivery group (n = 256) received the nutrition manual in the mail with no further information. Medical practices in the control group (n = 255) did not receive the nutrition manual.

Follow-up interviews with medical staff and observational assessments were conducted at four to six months after dissemination of the manual. Adherence scores were calculated for four areas: office organization, nutrition screening, nutrition advice or referral, and patient follow-up. There was low attendance at the workshop session; less than 50 percent of assigned practices sent representatives (120 of 244). The authors of the trial used an "intent to treat" approach for the primary statistical analyses and included all practices in the workshop group regardless of attendance. The workshop group was significantly more adherent to the manual's recommendations for office organization at follow-up than either the postal-delivery group (28.5 vs. 24.7 percent, p < 0.005) or the control group (28.5 vs. 23.0 percent, p < 0.001). Of those practices who sent a representative to the workshop, 30.6 percent were adherent to the recommendations for office organization. There was no significant difference between the postal-delivery group and the control group for office organization (24.7 vs. 23.0 percent).

The workshop group was also significantly more adherent to the manual's recommendation for nutrition screening than either the postal-delivery group (23.5 vs. 21 percent, p < 0.05) or the control group (23.5 vs. 20.5 percent, p < 0.05). Of those practices that sent a representative to the workshop, 25 percent were adherent to the nutrition screening recommendations. There was no significant difference between the postal-delivery group and the control group for nutrition screening (21 vs. 20.5 percent). There was no statistically significant difference between the three groups for providing nutrition advice (workshop 54.9 percent, postal delivery 53 percent, control 52.3 percent), nor for patient follow-up (workshop 14.6 percent, postal delivery 13.6 percent, control 13.6 percent). A secondary analysis showed that those practices who attended the workshop were significantly more likely than either the postal-delivery group (57 vs. 53 percent, p < 0.05) or the control group (57 vs. 52.3 percent, p < 0.05) to provide nutrition screening. There was no significant difference observed for patient follow-up on secondary analysis.

Training workshops appear to hold some promise as a dissemination strategy; however, motivating medical professionals to attend these sessions may be a difficult barrier to overcome. Further research in this area is needed.

#### Postal delivery

One RCT[17] evaluated the effectiveness of postal delivery as a dissemination strategy. This trial compared the effectiveness of postal delivery with a training workshop to disseminate an NCI nutrition manual to primary care practices. Postal delivery was not found to be an effective method to disseminate the nutrition manual. Please refer to the section above on Workshops for the detailed results of this study.

### Dissemination Studies That Targeted Worksites

#### Passive dissemination

The Working Well Trial[14,24] randomized 114 worksites of over 28,000 workers to test the effectiveness of health promotion activities that were planned and delivered with a high level of employee participation. The intervention phase lasted for two years, and then nutrition materials were disseminated to the control sites, followed by a further two-year assessment. The investigators were particularly interested to see if the control sites would utilize the materials. No information was given about the actual strategies used to get the nutrition intervention materials to the control group, nor was any report of measure of uptake given. No changes occurred in the level of nutrition activities in the control sites.

An opinion leader strategy was tested using peer educators in the worksite intervention called "5-A-Day: Healthier Eating for the Overlooked Worker". While rated methodologically weak, it holds promise as an area for further research. It was an RCTof 5-A-Day intervention to increase fruit and vegetable consumption in an ethnically mixed population of 2,091 lower socioeconomic and trade employees[9,10]. Both the intervention group and the control worksites received an 18-month intervention program of education materials through workplace mail, cafeteria promotions, and speakers. In the intervention group, naturally occurring work "cliques" were identified, and within those, ratings were given to each individual regarding their degree of "centrality" to communication ties and flow. Those rated highest in "centrality" became the peer educator for that clique, mimicking the "opinion leader" strategy.

Peer educators attended a 16-hour training program where they were given information about health benefits of eating fruits and vegetables, cultural trends in dietary practices, peer educator's roles and responsibilities, and five persuasive communication strategies (foot-in-the-door, fear appeal, benefits, peer pressure, and questioning) and ways to initiate informal conversations about fruits and vegetables. They were instructed to engage in nutrition education of the co-workers for about two hours per week, on work time. They also distributed 5-A-Day materials produced specifically for this population: a nine-booklet resource guide, four issues of a newsletter, enabling gifts such as a recipe book, and vegetable seeds. The peer educator intervention lasted nine months, with consumption measured at the end of the intervention and six-month follow-up.

The result was an increase in fruit and vegetable consumption of 0.77 total servings per day more in the intervention group compared with the controls (measured by recall, p < 0.001) and an increase of 0.46 total daily servings (measured by food frequency, p < 0.002)[9]. The effect was maintained at six-month follow-up for intake recall (increase of 0.41 daily servings, p = 0.034) but not for food frequency[9]. In analysis of the frequency and duration of peer-education contact with co-workers, greater contact with the peer educators was related to larger immediate increases in fruit and vegetable intake, particularly vegetable intake, but was not related to total intake at six-month follow-up[10]. A qualitative design, used to study the educational strategies used by the peer educators in the intervention group,[11] found that these studies differed by gender and ethnicity[11]. Hispanic educators were more likely to use individual, rather than group, change strategies than non-Hispanic educators; men more frequently used strategies such as "mock competition", "giving materials" and "encouragement", while female peer educators more often used "creating context", and "keeping 5-A-Day visible"[11].

Few worksite dissemination strategies have been evaluated. In one, the dissemination strategy was not evaluated[14]. The other study using an opinion leader strategy had at least a short-term impact on consumption.

### Dissemination Studies That Targeted Individuals

#### Media strategies

Two studies evaluated multiple media channels (print, television, and radio) to assess the impact of the media campaigns on telephone calls to an information telephone line[13,15]. "Project Lean" (Low-Fat Eating for America Now) was a three-year initiative, begun in 1989, to reduce dietary fat consumption. The media campaign led to hotline access of 300,000 consumer calls in 18 months (25,000 to 28,000 calls/month), but the calls declined as publicity declined, and the line was terminated due to expense, estimated to be US $300,000 per year[13]. While these outcomes were not assessed in a direct comparison, some important lessons were learned in this study:[13] that well-placed advertising may be the most appropriate and effective communications strategy for a national nutrition social marketing campaign as it can, more easily than Public Service Announcements (PSA's), be tailored to the particular audience; can communicate information more directly and can reduce the need for an information hotline or follow-up materials. Furthermore, building a network of state and local programs and partnerships with the food service industry allowed the campaign to reach a broader audience[13].

A second primary study was identified which was an analysis of calls to the Cancer Information Service (CIS) hotline. Callers were asked, "How did you first find out about the CIS?" Records of a subsample of people (214,472) who inquired about smoking, nutrition, Pap smears, and breast self-evaluation were reviewed. Television was the most frequently reported source of learning about the information line, regardless of age, gender, or ethnic group (except callers of Asian or Pacific heritage, who reported publications as the more common source of information about the hotline)[15].

The media dissemination strategies, particularly television messages, can make people aware of information lines and prompt them to call. However, from these two studies, it appears that the lines are expensive to advertise and maintain.

## Discussion and implications

There has been increased recognition of the need for processes to transfer new knowledge into routine practice. Traditional methods of knowledge transfer such as journals and conferences have not proven effective in changing behavior[25]. Emphasis has been placed on the importance of research examining the dissemination of evidence-based knowledge and its uptake by the targeted recipients. Target audiences include providers, policymakers and the general public.

There are several limitations of this review. It does not include the effectiveness of the dietary interventions themselves, but of the dissemination interventions to get others to know about the interventions. The results and conclusions are based on information available in published English-language reports. Contact with authors could have compensated for any reporting difficulties that resulted in a lower quality rating of the studies. Meta-analysis was deemed inappropriate due to the diversity in the target groups, interventions and outcome measures.

There are few studies of dissemination of dietary interventions for cancer prevention. Overall, the quality of the evidence is not strong and is primarily descriptive rather than evaluative. Either process measures (numbers of calls, numbers of physicians educated, or number of educations sessions held) are reported or outcomes are often non-validated self-report measures. Controlled studies need to be done for any dissemination strategies, and dissemination and diffusion strategies with different messages and different target audiences need to be compared. More studies of healthcare providers with strategies such as opinion leaders or academic detailing should be done. The idea of a peer educator who is identified more as an opinion leader warrants further exploration. Cost-effectiveness needs to be established for any interventions.

Most of the focus of research on healthy diet and cancer has been on evaluating interventions to promote behavior change. There is a lack of information on how to disseminate these findings to the community. Questions to address in future research include: What is the effectiveness of reminder strategies for health professionals to give interventions in-patient encounters? What innovative technologies can be brought to the dissemination strategies? Once media strategies have alerted the public to services, can effective interventions then be disseminated to individuals in such a way that they will utilize them to change dietary habits? Or is there an effective combination or sequencing of strategies that will result in dietary change? What policy level strategies are effective in at promoting dissemination of healthy diet interventions? What maintenance strategies can be incorporated to maintain the uptake and utilization of the evidence?

## Competing interests

The author(s) declare that they have no competing interests.

## Authors' Contributions

D. Ciliska: review of the literature, conceptualization, writing and editing

P. Robinson: review of the literature and comments on the draft

T. Armour: review of the literature and comments on the draft

P. Ellis: review of the literature and comments on the draft

M. Browers: review of the literature and comments on the draft

M. Gauld: review of the literature and comments on the draft

F. Baldassarre: review of the literature and comments on the draft

P. Raina: review of the literature and comments on the draft

## Supplementary Material

Additional File 1 This table details the included studies in this review: citation, design, methodologic quality rating, strategy evaluated and findings. Click here for file
