# Supplementary material for: Diffusion and dissemination of evidence-based dietary srategies for the prevention of cancer
Source: Nutr J. 2005 Apr 8;4:13. doi: 10.1186/1475-2891-4-13 (PMC1087879; doi:10.1186/1475-2891-4-13)
Supplement: Additional File 1 — This table details the included studies in this review: citation, design, methodologic quality rating, strategy evaluated and findings. [file 1475-2891-4-13-S1.doc]

| Table 1: Strategy for dissemination of cancer control interventions in adult healthy diet | | | | |
| --- | --- | --- | --- | --- |
| **Author (Year)**  **Country**  **Study purpose** | **Study design**  **Target group**  **Quality Assessment** | **Dissemination strategy evaluated** | **Intervention(s)** | **Findings** |
| Albright, CL[12] (1992)  United States  Purpose:  To test a dissemination model for providing clinical preventive medicine (CPM) training to internal medicine faculty across the US. | Study Design:  Cohort, one group, pre/post  n = 10 internists were trained as “trainers”  91 faculty members attended home-site seminars led by trainers  Target Group: General Internal Medicine faculty in the US  Quality Assessment: Weak | Train-the-Trainer  (Month-long training, off-site) | CPM curriculum encompassed: smoking cessation, clinical nutrition, weight control exercise, hyperlipidemia, and national screening guidelines.   - curriculum provided content on clinical teaching and medical decision-making - clinical nutrition section included interventions that promoted adult healthy diets | - Fidelity ratings obtained from videotapes of the home-site seminars given by the trainers indicated that the trainers adhered closely to the CPM - Trainers teaching home-site internal medicine faculty: knowledge of smoking cessation, cholesterol and screening-specific information of faculty members significantly increased post-seminar in the last 2 of the 3 years studied (p<0.02 and p<0.001) - faculty’s ratings of their self-efficacy to implement the CPM practices for clinical nutrition demonstrated significant post-test increases for all 3 years (p<0.0001) - faculty use of specific behavior-change interventions to promote healthy diet increased (diaries, self-help materials, and social support) (p<0.05) - 85% of the faculty in 1986, 96% in 1987, and 84% in 1988 reported teaching the CPM strategies to their house staff - House staff reported significant increases in the degree to which the faculty addressed CPM in their teaching interactions (1987: p<0.0001 and 1988: p<0.04) - House staff ratings of their self-efficacy to implement specific preventive medicine strategies increased in both years (1987: p<0.0001 and 1988: p<0.002) |
| Anderson, DM[15] (1989)  United States  Purpose:  To examine inquiries received by the CIS, a telephone hot-line, to determine:  1) effects of different media in stimulating calls to the CIS, and  2) demographic characteristics of callers in 4 cancer prevention and early detection subjects: smoking, nutrition, Pap smear screening and breast self-examination | Study Design: Descriptive study  Retrospective analysis of 5 years of inquiries to one national and 26 local CIS offices in 4 subject areas. - standardized call record form was completed for each call.  - Demographic information was only collected during the last 2 years of the study for first-time, non-health professional callers and was limited by federal stipulations to 20% of callers in 5 CIS offices  n = 57,374 nutrition- related calls over the five years studied  Target Group:  Smokers in the US, Mexico and other countries  Quality Assessment: Weak | Multiple media sources (television, radio, and newspapers) | CIS – a telephone based information and education program of the National Cancer Institute. | - source of callers' learning about the CIS hot-line were: television (33.9%), radio (28.2%), publications (included newspapers, magazine, pamphlets and posters) (26.9%), health care providers (4.3%), significant others (4.6%); and telephone assistance (2.2%) - television was the most common information source reported by callers for both sexes (72.2% male callers and 60.7% of female callers) - There was an inverse relationship between frequency of television cited as an information source and callers age and education. In the 19-year old or less age group 81.7% of callers cited television compared to 39.6% of callers in the 60-year or older age group - Television was the predominant source for 4 of the 5 ethnic groups– Caucasians, African-Americans, Hispanics, and Native Americans. For callers of Asian or Pacific Island heritage, the most frequently cited source was publications (46.7%), followed by television (32.1%). |
| Buller, DB[9] (1999)  United States  Purpose:  To test a peer-education strategy to promote the 5-a-Day message.  Related Papers:  Buller, DB[10] (2000)  (reported below)  Larkey, LK[11] (1999) (reported below) | Study Design: RCT  Experimental group:  n = 505 employees (in 46 cliques) assigned to receive 5-a-Day peer-education plus the general 5-a-Day program  Control group:  n = 492 employees (in 46 cliques) assigned to receive the general 5-a-Day program (cafeteria promotions and workplace mail) only  Target Group: Anglo and Hispanic worksite population in the US (lower socioeconomic labor and trade employees)  Quality Assessment: Weak | Peer-educators (n=42)  (Chosen for ‘centrality’, rated highest by peers in communication ties and flow)   - attended a 16-hour training program: training in persuasive communication techniques - expectation was that they would spend 2 hours/week discussing fruit and vegetable intake with coworkers | 5-a-Day message plus accompanying 5-a-Day printed materials | Immediate changes post-intervention in awareness, attitudes, and dietary behavior:   - Employees receiving peer- education increased their awareness of the 5-a-Day program (p<0.001) - knowledge of the 5-a-Day concept, attitudes toward fruit and vegetable intake (p=0.024 - <0.001) - number of daily servings of fruits and vegetables consumed (Increase of 0.77 on 24-hour intake recall p<0.001 and 0.46 on food frequency questionnaire items p=0.002)   Persistence of changes in awareness, attitudes, and dietary behavior (6-month followup):   - general persistence of the statistically significant increases in the peer-education group, but of reduced magnitude for knowledge of the 5-a-Day program and diet-related attitudes - statistically significant increases total number of daily servings persisted when measured by 24-hour intake recall (0.41, p=0.034), but not by food frequency questionnaire |
| Buller, DB[10] (2000)  United States    Related Papers:  Buller, DB[9] (1999)  (reported above)  Larkey, LK[11] (1999) (reported below) | Same study as above | Same study as above | Same study as above | Findings pertain only to the peer-education (experimental group)   - 57% of employees reported printed materials stimulated discussion of fruits and vegetables with co-workers during program, 31% still discussing 6 months later, 69% discussed printed material with a family member during intervention - greater contact with peer- educators was related to larger immediate increases in total consumption of fruits and vegetables (p=0.003) as measured by food frequency items - when food types were examined separately, peer-educator contact was positively related to immediate increased vegetable intake (p=0.002), but not to fruit or juice intake - the more employees reported reading the printed material, the smaller the observed immediate increase in fruit consumption (p=0.002) - there was no significant association between peer contact and changes in total intake of these foods at the 6-month followup |
| Dietrich, AJ [16] (1992)  United States  Purpose:  To test the impact of physician education and facilitator assisted office-system interventions on cancer early detection and preventive services | Study Design: RCT   - 98 of the 102 practices that agreed to participate completed the study. - unit of randomization was the practice as represented by one physician.   Four groups:  Facilitator only:  n = 24 practices  Workshop + Facilitator:  n = 26 practices  Workshop only:  n = 24 practices  Control:  n = 24 practices;  Target Group:  Office-based GPs and general internists in New Hampshire and Vermont.  Quality Assessment: Weak | (1) Facilitators: Visited each practice 3-4 times over 3 months x approximately 120 minutes each.  - Performed an initial audit of each practice to assess the status of preventive care and assisted practices in the design and implementation of office system interventions. Practices only implemented those interventions that meet their perceived needs  (2) Facilitator + workshop  Same as (1) plus physician from each practice attended a 1-day workshop led by an expert who reviewed NCI’s prevention and screening recommendations and taught specific skills. Also provided a written syllabus  Note: The workshop only and the control groups did not receive information on the use of office-systems interventions for cancer prevention or early detection | Multiple office-system interventions including preventive care flow sheets, chart stickers, health education posters and brochures, and patient health diaries  (none of the interventions were computer-based) | response rate for the cross-sectional survey was 91% (n = 2436 patients) pre-experiment and 93% (n = 2595) at 12-month follow-up.   - more eligible patients in the Facilitator Only group reported their physician had advised them to reduce fat intake compared to patients in the control group at 12-month follow-up (proportion 0.56 vs. 0.47, p<0.05, baseline results were used as covariates). - *no* significant increase in the number of eligible patients in the facilitator + workshop group reporting their physician had advised them to reduce fat intake compared with patients in the control group at 12-month follow-up (proportion 0.51 vs. 0.47) - *no* significant increase in the number of eligible patients in the facilitator only or facilitator + workshop groups reporting their physician had advised them to increase fibre consumption compared to patients in the control group at 12-month follow-up (Proportion 0.48 (facilitator only) vs. 0.38 (control); Proportion 0.41 (facilitator + workshop) vs. 0.38 (control))   Report’s overall conclusion: Community practices assisted by a facilitator in the development and implementation of an office system can substantially improve provision of cancer early detection and preventive services |
| Larkey, LK[11] (1999)  United States  Purpose: To assess which persuasive strategies are used by peer-health educators, which strategies are used more in one-on-one vs. group contexts, and which strategies are most likely to be used by males and by females  Related Papers:  (1) Buller, DB[10] (2000)  (Same project, see additional findings above)  (2) Buller, DB[9] (1999)  (Same project, see additional findings above) | Study Design:  Qualitative, descriptive study of peer-educator within original randomized trial  Target Group:  Anglo and Hispanic worksite population in the US  Quality Assessment: Weak | Peer-educators (n = 42)  (Chosen for ‘centrality’, rated highest by peers in communication ties and flow) | 5-a-Day message (to increase fruit and vegetable intake) plus accompanying 5-a-Day printed materials | Peer-health educators were more likely to use “role modeling” (p=0.0004) and “creating context” (p<0.0001) as collective (group) change strategies, while “encouragement” (p=0.0009) and “responding to employee needs” (p=0.0001) were more likely to be used as individual change strategies  Strategies used differed by gender. “Mock competition,” “giving materials,” and “encouragement” were used by men significantly more than “creating context” and “keeping 5-a-Day visible”. Women used “creating context” and “keeping 5-a-Day visible” significantly more than “mock competition”, “giving materials” and “encouragement” (p<0.0001 for all contrasts)  Hispanic peer health educators were more likely to use individual change strategies than their non-Hispanic counterparts (p=0.0128) |
| Patterson, RE[14] (1998)  United States  Purpose:  To test whether the Working Well Trial nutrition intervention activities were maintained after the research program or were adopted by control sites.  Related Papers:  Sorensen, G[24] (1998)  Dissemination of worksite smoking cessation interventions from the Working Well Trial to control cited at the end of the trial. | Study Design: RCT  n = 54 control sites  Target Group: Worksite management  Quality Assessment: Moderate | Intervention materials were given to control sites (method not specified) at the conclusion of the Working Well Trial | Nutrition interventions from the Working Well Trial | Only the findings pertaining to the control sites at the conclusion of the Working Well Trial are reported in this table.   - there was a significant increase in nutrition activity score (composite of nutrition classes or weight loss programs; self-help nutrition manuals and guides; videotapes, posters or brochures related to nutrition) from baseline to the end of the Working Well trial (2-year interval between start and completion of the trial) (p=0.0012) - there was no significant increase on nutrition activity score in the control sites, between the end of the Working Well trial (point of dissemination of the nutrition interventions to control sites) and at the follow-up survey conducted 2 years - at follow-up, there was no significant difference between nutrition activity scores in the intervention compared with the control worksites |
| Samuels, SE[13] (1993)  United States  Purpose:  To report on the first 3 years of the Project LEAN campaign, a national social marketing intervention, designed to promote dietary change. | Study Design:  One group, post-intervention, process evaluation  Target Group:  General adult population  Quality Assessment: Weak | Media awareness campaign (television and print media) | Telephone hot-line which provided advice and offered an information booklet to callers | - as a result of the ads and campaign publicity, the hot-line received nearly 300,000 consumer calls - during the first 12 months of the campaign, calls peaked at 25,000 to 28,000 a month - as publicity declined, so did calls to the hot-line - Hot-line terminated at end of 18 months due to expense (over $300,000 per year) |
| Tziraki, C (2000)[17]  United States  Purpose:  To determine the effectiveness of two strategies for promoting the use of an NCI nutrition manual by primary care physicians and their office staff | Study Design:  RCT   - 810 practices were randomized; 55 practices had a change in status and became ineligible after randomization   Workshop group: n = 244 practices received the manual and were invited to a training workshop  Postal delivery group: n = 256 practices were mailed the manual  Control group: n = 255 practices; did not receive the manual  Target Group:  Free-standing primary care practices in Pennsylvania and New Jersey  Quality Assessment: Weak | (1) Workshop  (One staff member from each practice was invited to attend a 3-hour training session. Training was provided in the 4 major components of the manual; how to organize the office environment; how to screen patient adherence; how to provide dietary advice; and how to implement a patient follow-up system)  (2) Postal delivery of the manual only (no training) | NCI sponsor Nutrition Manual for cancer prevention (contains multiple interventions)   - Modeled after the NCI publication: “How to help your patients stop smoking.” - The manual addressed brief counseling techniques, office system organization, material resources, staff training, and patient educational materials | Follow-up interviews and observational assessments were conducted at 4 - 6 months post-dissemination with a physician and staff member from each practice. Adherence scores were calculated for 4 areas: office organization, nutrition screening, nutrition advice or referral, and patient follow-up   - < 50% of practices assigned to the workshop group sent representatives to the training workshop (120 of 244) - workshop group was significantly more adherent to the manual’s recommendations for office organization at follow-up than the Postal-Delivery group (28.5% vs. 24.7%, p<0.005) and Control group (28.5% vs. 23.0%, p<0.001); these analyses included all practices in the Workshop group regardless of attendance at the training session. - Of those practices who attended the workshop, 30.6% were adherent to the recommendations for office organization - Workshop group was significantly more adherent to the manual’s recommendations for nutrition screening at follow-up than the Postal Delivery group (23.5% vs. 21%, p<0.05) and Control group (23.5% vs. 20.5%, p<0.05). - Of those practices attending the workshop, 25% were adherent to the recommendations for nutrition screening - no significant difference between the Postal delivery and control groups for office organization (24.7% vs. 23.0%) or nutrition screening (21% vs. 20.5%) - no statistically significant difference between the 3 groups for nutrition advice (Workshop 54.9%; Post 53%; Control 52.3%) nor for patient follow-up (Workshop 14.6%; Postal Delivery 13.6%; Control 13.6%). - the attending workshop practices were significantly more likely than either Postal Delivery (57% vs. 53%, p<0.05) or Control groups (57% vs. 52.3%, p<0.05) to provide nutrition screening |
